# Mucosal Vaccine Development: From Adjuvant Design to Next-Generation Delivery Strategies

**DOI:** 10.3390/biomedicines14051060

**Published:** 2026-05-07

**Authors:** Wook-Heon Lee, Eunsoo Kim

**Affiliations:** College of Pharmacy and Research Institute for Drug Development, Pusan National University, Busan 46241, Republic of Korea; luh0210@naver.com

**Keywords:** mucosal vaccine, secretory IgA, mucosal adjuvant, intranasal immunization, sublingual vaccination, needle-free vaccine

## Abstract

Most infectious pathogens enter the host through mucosal surfaces, yet conventional injectable vaccines primarily induce systemic immunity without eliciting robust secretory immunoglobulin A (SIgA) responses at mucosal sites. The COVID-19 pandemic highlighted this limitation, as intramuscular mRNA vaccines failed to establish durable mucosal immunity in the upper respiratory tract. This review covers recent progress in mucosal vaccine development. We first discuss the organization of the mucosal immune system, focusing on SIgA induction, tissue-resident memory T ($T_{\mathrm{RM}}$) cells, and resident memory B ($B_{\mathrm{RM}}$) cells. We then examine mucosal adjuvants, from cholera toxin and heat-labile enterotoxin derivatives to stimulator of interferon gene (STING) agonists and a strategy to enhance alum adjuvanticity through neutrophil elastase inhibition. Delivery routes including intranasal, oral, and sublingual administration are reviewed alongside viral vectors, nanoparticles, mRNA-lipid nanoparticles, virus-like particles, and engineered bacterial platforms. The roles of innate immune cells, T helper cell subsets, and the microbiota in shaping vaccine responses are discussed. Finally, we survey licensed mucosal vaccines and the COVID-19 mucosal vaccine pipeline, analyze persistent barriers to clinical translation including the absence of validated mucosal correlates of protection, and outline future directions for thermostable formulations and systems biology-driven vaccine design.

## 1. Introduction

Mucosal surfaces, including the gastrointestinal, respiratory, and urogenital tracts, represent the primary portals of entry for the vast majority of human pathogens [1,2]. These surfaces are protected by the mucosal immune system, which functions largely independently of systemic immunity [3]. Secretory immunoglobulin A (SIgA) serves as the principal effector antibody at mucosal sites [3]. SIgA neutralizes pathogens and toxins at mucosal surfaces before they breach the epithelial barrier, a process termed immune exclusion [4]. Despite this, most licensed vaccines are administered by injection and primarily induce systemic IgG responses, offering limited protection at mucosal surfaces where infection begins [5,6].

This gap between systemic and mucosal immunity directly limits vaccine efficacy against respiratory pathogens. The COVID-19 pandemic illustrates this clearly: intramuscular mRNA vaccines effectively prevented severe disease but failed to induce durable mucosal immunity, leaving the upper respiratory tract vulnerable to infection and transmission [7,8]. The emergence of immune-evasive variants has further highlighted the need for mucosal vaccines capable of blocking infection at the point of entry [9,10].

Mucosal vaccine development has been pursued for decades. Early successes such as the oral polio and rotavirus vaccines demonstrated its feasibility [11] yet only a handful of mucosal vaccines have been licensed to date. Key challenges include the need for safe mucosal adjuvants, antigen degradation at mucosal surfaces, and the inherent tolerogenic environment that can suppress rather than promote immune responses [12,13]. Over the past two decades, however, advances in mucosal immunology have yielded new adjuvant and delivery strategies. More recently, engineered probiotic bacteria have emerged as oral delivery vehicles that can produce and release antigens directly in the gut [14,15].

This review covers recent progress in mucosal vaccine development. We first review the structural and functional organization of the mucosal immune system, focusing on the mechanisms governing SIgA induction. We then discuss the evolution of mucosal adjuvants, from classical enterotoxin-based adjuvants to novel molecular strategies. Diverse delivery routes and platforms, including intranasal, oral, sublingual, viral vector-based, and engineered bacterial approaches, are examined. The roles of innate immune cells, T helper cell subsets, and the microbiota in shaping mucosal vaccine responses are discussed. Finally, we survey the current landscape of approved and clinical-stage mucosal vaccines and highlight the key challenges and future directions for the field.

## 2. Mucosal Immune System: Structural and Functional Basis for Vaccination

### 2.1. Mucosal-Associated Lymphoid Tissues

**Figure 1:**
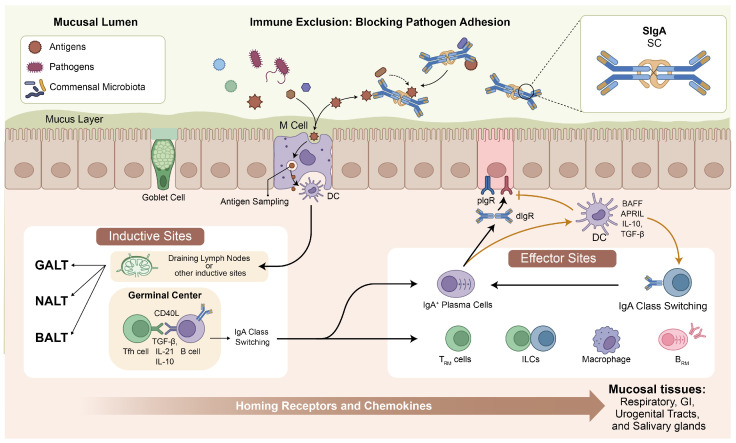
Overview of the mucosal immune system. Antigens are sampled by microfold (M) cells and delivered to dendritic cells (DCs) in mucosal-associated lymphoid tissues (GALT, NALT, BALT). T-dependent IgA class switching occurs in germinal centers via T follicular helper (Tfh) cell-B cell interactions, while T-independent switching is driven by BAFF and APRIL from epithelial cells and DCs. Dimeric IgA is transported across the epithelium by the polymeric immunoglobulin receptor (pIgR) and released as secretory IgA (SIgA) to mediate immune exclusion. Effector sites harbor $T_{\mathrm{RM}}$ cells, $B_{\mathrm{RM}}$ cells, innate lymphoid cells (ILCs), and macrophages. The common mucosal immune system (CMIS) directs immune cell migration to distant mucosal tissues.

The mucosal immune system is organized into inductive sites, where immune responses are initiated, and effector sites, where effector cells and antibodies carry out protective functions [1,2] (Figure 1). The best-characterized inductive sites include the gut-associated lymphoid tissues (GALTs), comprising Peyer’s patches (PPs), isolated lymphoid follicles, and the appendix [16]. The nasopharynx-associated lymphoid tissues (NALTs), including the palatine tonsils and adenoids, serve as the primary inductive sites in the upper respiratory tract [17]. Bronchus-associated lymphoid tissues (BALTs) are also found in the respiratory tract, although these structures are less consistently present in humans than in experimental animals [18]. These lymphoid structures contain specialized microfold cells (M cells) that sample luminal antigens and deliver them to underlying dendritic cells (DCs) and macrophages to initiate adaptive immune responses [19].

Different inductive sites contribute distinct immunological functions. The tonsils, the human equivalent of NALT, function as both inductive and effector sites, supporting IgA and IgG responses and sharing functional features with GALT [20]. Intranasal immunization with mucosal adjuvants such as cholera toxin (CT) induces strong immune responses in both external secretions and systemic compartments, often exceeding those achieved by oral delivery [17,21,22]. Both routes offer distinct advantages depending on the target pathogen and the mucosal sites requiring protection. Oral immunization preferentially induces SIgA in the gastrointestinal tract, while intranasal immunization elicits broader mucosal responses across the respiratory and genital tracts [21,23].

The effector sites of the mucosal immune system include the lamina propria of the intestinal, respiratory, and urogenital tracts, as well as exocrine glands such as salivary and lacrimal glands [1]. At these sites, IgA-secreting plasma cells produce dimeric or polymeric IgA, which is transported across epithelial cells by the polymeric immunoglobulin receptor (pIgR) and released into the lumen as SIgA [3,24]. These sites are interconnected through the common mucosal immune system (CMIS), which enables immune responses initiated at one mucosal site to confer protection at distant surfaces. The strength of this cross-protection depends on the route of immunization [21,23].

### 2.2. Secretory IgA: Structure, Function, and Regulation

SIgA is the most abundantly produced immunoglobulin in the human body, with an estimated daily production of 3–5 g at mucosal surfaces [3]. SIgA consists of two IgA monomers linked by a joining (J) chain and a secretory component (SC) derived from the polymeric immunoglobulin receptor (pIgR) during epithelial transcytosis. The SC confers resistance to proteolytic degradation at mucosal surfaces [3,4]. SIgA mediates immune exclusion by preventing pathogen adherence, neutralizing toxins and viruses, and modulating the commensal microbiota [4,25].

Early studies identified IL-5 and IL-6 as key Th2-type cytokines driving IgA plasma cell differentiation [26], with IL-10 also promoting IgA synthesis, particularly in humans [27]. These cytokines are produced at high frequency by Th2 cells in mucosal effector sites such as the intestinal lamina propria [26]. However, Th1-promoting adjuvants such as IL-12 can also support mucosal SIgA induction, indicating that SIgA is not exclusively dependent on Th2 responses [28,29].

SIgA production is regulated through both T-dependent and T-independent mechanisms [6,27]. T-dependent IgA class switching occurs in the germinal centers of Peyer’s patches and NALT, where follicular helper T (Tfh) cells provide help to B cells through CD40L-CD40 interactions [30]. Key cytokines driving this process include TGF-β, IL-21, and IL-10 [31]. T-independent IgA responses, in contrast, are driven by innate signals from epithelial cells and dendritic cells in response to microbial stimulation. Epithelial cells and DCs produce BAFF (B cell-activating factor) and APRIL (a proliferation-inducing ligand), along with TGF-β and IL-10, which promote IgA class switching and plasma cell differentiation in the lamina propria [6,32,33]. The interplay between these T-dependent and T-independent pathways determines the magnitude and quality of SIgA responses to both commensal and pathogenic microbes, with direct implications for adjuvant selection in mucosal vaccine design [6].

### 2.3. Tissue-Resident Memory Cells at Mucosal Surfaces

Beyond SIgA, tissue-resident memory cells provide a critical layer of mucosal defense against reinfection. Tissue-resident memory T ($T_{\mathrm{RM}}$) cells are non-circulating memory T cells that permanently reside in peripheral tissues, including mucosal surfaces [34,35]. Upon antigen re-encounter, $T_{\mathrm{RM}}$ cells rapidly produce cytokines and chemokines that recruit immune cells and reinforce barrier defense [36]. Both ${\mathrm{CD4}}^{+}$ and ${\mathrm{CD8}}^{+}$ $T_{\mathrm{RM}}$ cells have been identified in the respiratory mucosa, where they contribute to protection against influenza, RSV, and SARS-CoV-2 [37,38]. Mucosal vaccination is particularly effective at generating $T_{\mathrm{RM}}$ cells at respiratory surfaces, whereas systemic vaccination primarily induces circulating memory cells with limited capacity to control pathogens at mucosal entry sites [39,40].

Tissue-resident memory B ($B_{\mathrm{RM}}$) cells represent a complementary arm of local mucosal memory. $B_{\mathrm{RM}}$ cells persist in the lungs following respiratory infection and, upon rechallenge, rapidly increase their migratory capacity, localize to infected sites through CXCR3-dependent chemotaxis, and differentiate into antibody-secreting plasma cells that deliver antibodies directly to sites of viral replication [41,42]. This spatially targeted antibody response provides a speed and precision that circulating memory B cells cannot match. Together, $T_{\mathrm{RM}}$ and $B_{\mathrm{RM}}$ cells are now considered key correlates of protection for mucosal vaccines, and optimizing their generation through adjuvant and delivery design remains a priority [43,44].

## 3. Mucosal Vaccine Adjuvants: From Classical Toxins to Novel Molecular Adjuvants

**Figure 2:**
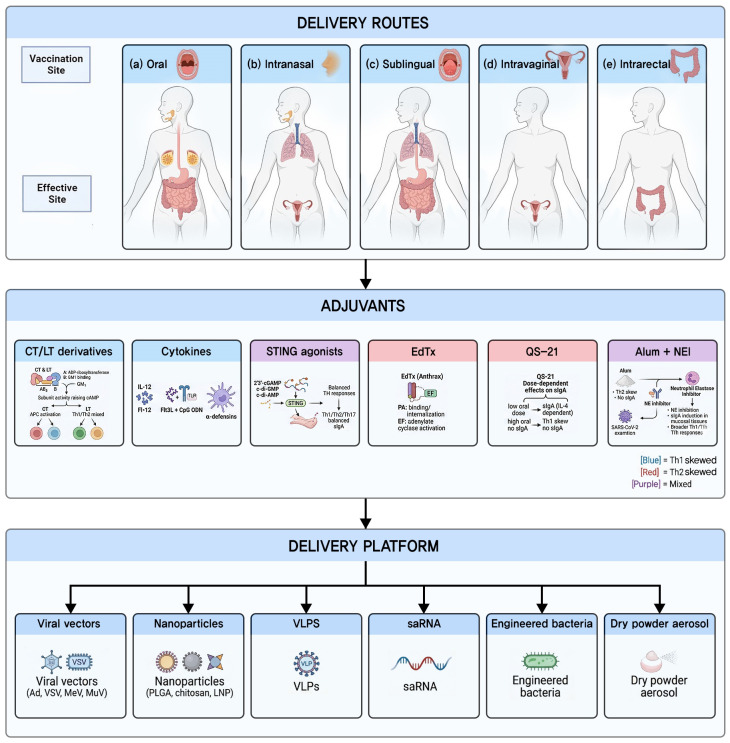
Mucosal vaccine delivery routes, adjuvants, and platforms. (**top**) Delivery routes (oral, intranasal, sublingual, intravaginal, and intrarectal), each targeting distinct inductive and effector sites. (**middle**) Adjuvant classes with color-coded T helper polarization: blue, Th1-skewed; red, Th2-skewed; purple, mixed. (**bottom**) Delivery platforms including viral vectors, nanoparticles, virus-like particles (VLPs), self-amplifying RNA (saRNA), engineered bacteria, and dry powder aerosol.

Identifying safe and effective mucosal adjuvants remains a major challenge in the field. Unlike the systemic immune compartment, mucosal surfaces are inherently tolerogenic, and antigens delivered without appropriate adjuvant signals are more likely to induce tolerance than protective immunity [12,13]. Mucosal adjuvants must therefore enhance the magnitude of immune responses and direct their quality, including SIgA induction, T helper cell polarization, and $T_{\mathrm{RM}}$ generation [6,45] (Figure 2).

### 3.1. Enterotoxin-Based Adjuvants: Cholera Toxin, Heat-Labile Enterotoxin, and Their Derivatives

CT and the closely related heat-labile enterotoxin (LT) of *Escherichia coli* are the best-characterized mucosal adjuvants [45,46]. Both are AB5-type toxins composed of an enzymatically active A subunit and a pentameric B subunit that binds to GM1 ganglioside on host cell surfaces. The A subunit possesses ADP-ribosyltransferase activity that elevates intracellular cAMP, leading to activation of antigen-presenting cells, enhanced antigen uptake and processing, and upregulation of co-stimulatory molecules [46,47]. CT preferentially promotes Th2-type immune responses, characterized by IL-4 production and strong SIgA and serum IgG1 responses [48,49]. In contrast, LT induces a broader mixed Th1/Th2 cytokine profile, including IFN-gamma, IL-5, IL-6, and IL-10, making it more verstile for vaccines requiring balanced immunity [50]. CT also stimulates IL-1 and IL-6 secretion from APCs and epithelial cells, promoting B cell differentiation and IgA class switching [47]. These cytokines are further discussed as independent mucosal adjuvants in Section 3.2.

A key study demonstrated that the B subunit, rather than the ADP-ribosyltransferase activity, directs ${\mathrm{CD4}}^{+}$ T cell polarization by CT and LT [51]. Using chimeric toxins bearing the A subunit of one toxin and the B subunit of another, the authors showed that the B subunit determines whether the response is Th1- or Th2-biased while retaining mucosal adjuvanticity [51]. This finding enables the rational engineering of enterotoxin-based adjuvants with defined immune profiles. A second generation of double mutant cholera toxin adjuvants further improved safety by maintaining adjuvanticity without intracellular trafficking [52].

Despite their potency, the inherent toxicity of native CT and LT has precluded their clinical use in humans. This has driven the development of non-toxic derivatives, including genetically detoxified mutants such as CTS106 (mCT) and LTR192G/L211A (dmLT) [53,54]. These mutants retain adjuvant activity with greatly reduced toxicity. dmLT has advanced to clinical trials and enhances both mucosal and systemic immune responses to co-administered antigens [54,55].

### 3.2. Cytokines as Mucosal Adjuvants

Cytokines can serve as mucosal adjuvants to direct the quality and magnitude of immune responses. The nasal delivery route results in low but biologically active serum cytokine levels, making this approach safer than parenteral cytokine administration [56]. Interleukin-12 (IL-12) is an effective mucosal adjuvant that promotes both systemic and mucosal antibody responses to nasally co-administered antigens through ${\mathrm{CD4}}^{+}$ Th1-dependent mechanisms [28,29]. Combining IL-12 with CT redirected CT-induced Th2 responses toward a more balanced Th1 profile [57]. Nasal co-administration of IL-12 and CT predominantly induced Th1 responses, whereas oral co-administration favored Th2 responses, indicating that the delivery route determines the outcome of combination adjuvant strategies [57].

Not all cytokines promote mucosal immunity equally. Nasally administered IL-1 enhanced SIgA responses, whereas IL-6 promoted systemic immunity but failed to induce mucosal IgA [28]. This distinction underscores the importance of adjuvant selection for achieving the desired balance of mucosal and systemic protection. Other growth factors, including granulocyte-macrophage colony-stimulating factor (GM-CSF) and Flt3 ligand (Flt3L), expand dendritic cell populations at mucosal sites and enhance mucosal immune responses [58,59]. The combination of Flt3L cDNA and CpG oligodeoxynucleotides (CpG ODN) has been developed as a double adjuvant system targeting nasal dendritic cells, with enhanced mucosal immunity demonstrated in aging mouse models [58,59].

Beyond classical cytokines, chemokines and antimicrobial peptides have also been investigated for their mucosal adjuvant properties. Lymphotactin, RANTES, and defensins exert adjuvant activity for systemic immunity when nasally administered with antigens [56]. Defensins are of particular interest as they function both as antimicrobial effectors and modulators of adaptive immunity. α-Defensins recruit and activate dendritic cells and enhance antigen uptake, bridging innate and adaptive immune responses at mucosal surfaces.

The concept of combination adjuvant strategies, in which two or more adjuvants with complementary mechanisms of action are used together, has proven particularly effective for mucosal vaccination. The double adjuvant system of pFL and CpG ODN exemplifies this approach: pFL expands dendritic cell populations at mucosal sites while CpG ODN activates innate immunity through Toll-like receptor (TLR9) signaling [55,56,57]. The synergistic interaction between these two adjuvants results in enhanced and more durable mucosal immune responses than either adjuvant alone, particularly in the context of immunosenescence.

### 3.3. STING Agonists and Cyclic Dinucleotides

The stimulator of interferon genes (STINGs) pathway is an increasingly studied target for mucosal adjuvant development. Cyclic dinucleotides, including cyclic di-AMP (c-di-AMP), cyclic di-GMP (c-di-GMP), and 2′3′-cyclic GMP-AMP (cGAMP), are potent STING activators that promote robust innate and adaptive immune responses [60,61]. These molecules demonstrate strong mucosal adjuvant activity across multiple delivery routes, inducing balanced Th1/Th2/Th17 responses alongside SIgA production [61].

Sublingual targeting of STING with 3′3′-cGAMP was shown to promote both systemic and mucosal immunity against anthrax toxins [62]. This study established the sublingual route as viable for STING-mediated mucosal vaccination and demonstrated that STING agonists could replace traditional enterotoxin-based adjuvants for inducing protective mucosal immunity [62]. TING agonists also offer practical advantages: they can be manufactured synthetically, avoiding the production and safety issues associated with protein-based adjuvants.

### 3.4. Bacterial Toxins as Unconventional Adjuvants

Beyond CT and LT, other bacterial toxins have been investigated as mucosal adjuvants. The edema toxin (EdTx) of *Bacillus anthracis*, composed of protective antigen (PA) and edema factor (EF), is one such candidate. EdTx acts as a mucosal adjuvant when co-administered nasally with vaccine antigens at non-toxic doses, inducing both mucosal SIgA and systemic IgG antibodies with balanced Th1/Th2 cytokine profiles [63]. Subsequent studies dissected the individual contributions of PA and EF to EdTx’s adjuvant activity. PA facilitates cell binding and internalization of EF, while EF’s adenylate cyclase activity modulates intracellular signaling in antigen-presenting cells [64]. These findings broaden the available mucosal adjuvant repertoire and provide mechanistic insight into how bacterial toxins can enhance mucosal immunity against both homologous and heterologous antigens.

### 3.5. Saponin-Based Adjuvants

QS-21, a triterpene glycoside derived from Quillaja saponaria, has been widely used as a systemic adjuvant [65] and is also effective as an oral mucosal adjuvant [66]. A dose-dependent effect was observed: low oral doses of QS-21 promoted mucosal SIgA responses, while higher doses favored Th1-type responses without inducing SIgA [66]. This indicates that adjuvant dose optimization is critical for balancing mucosal and systemic immunity. Early IL-4 help was found to be essential for mucosal and systemic immunity induced by oral QS-21, further underscoring the role of Th2 cytokines in SIgA induction [66].

### 3.6. Enhancing Conventional Adjuvants: The Elastase Inhibition Strategy

Aluminum salts (alum) are the most widely used adjuvants in licensed injectable vaccines worldwide. However, alum primarily promotes Th2-type responses, is poor at inducing Th1 and cell-mediated immunity, and fails to induce SIgA at mucosal surfaces [67,68]. These limitations have largely excluded alum from mucosal vaccine applications.

We explored whether the adjuvanticity of alum could be enhanced through pharmacological inhibition of neutrophil elastase [69]. Alum stimulates the recruitment of myeloid cells, including neutrophils, to the injection site. We hypothesized that neutrophil-derived elastase might limit optimal immune responses to alum-adjuvanted vaccines. Indeed, mice co-administered with a pharmacological elastase inhibitor, or genetically lacking elastase, developed high-affinity serum IgG and IgA antibodies, as well as broader ${\mathrm{CD4}}^{+}$ T cell responses, including enhanced Th1 and T follicular helper cell responses [69]. This strategy also induced SIgA responses in mucosal tissues, which alum alone does not achieve [69]. When applied to the SARS-CoV-2 spike protein, the approach promoted both systemic and mucosal immunity, suggesting that it could broaden the protective efficacy of alum-based vaccines [69]. Alum-based vaccines are inexpensive and cold-chain-independent, making this strategy relevant for resource-limited settings. This approach complements other strategies to broaden alum-induced immunity, such as the combination of alum with TLR agonists or saponin-based adjuvants, although the elastase inhibition strategy is unique in its ability to induce mucosal SIgA from a parenterally administered vaccine.

## 4. Mucosal Vaccine Delivery Routes and Strategies

The route of mucosal vaccine delivery determines the anatomic distribution and magnitude of immune responses, as different routes target distinct inductive sites and generate preferential responses at specific effector sites [1,21] (Figure 2). Although mucosal vaccines can also be delivered via intravaginal and intrarectal routes, this review focuses on the intranasal, oral, and sublingual routes that are most advanced in clinical development. Route selection must balance immunological factors with practical considerations including patient compliance, ease of administration, and formulation stability.

### 4.1. Intranasal Vaccination

The intranasal route is among the most advanced approaches for mucosal vaccine delivery. Intranasal immunization directly targets the NALT, the primary inductive site for upper respiratory tract immunity [17,22]. When combined with mucosal adjuvants, intranasal antigens induce SIgA in the respiratory tract, salivary glands, and distant mucosal sites including the urogenital tract, as well as systemic IgG and cellular responses [21,22]. Nasal immunization with the protective antigen (PA) of *Bacillus anthracis* and mucosal adjuvants induced neutralizing antibodies and balanced Th cell responses, establishing that intranasal delivery can generate both mucosal SIgA and systemic protection [70].

Intranasal delivery has advanced rapidly in COVID-19 vaccine development, with several candidates reaching clinical stages or receiving emergency use authorization (EUA). The Chinese dNS1-RBD vaccine, based on a live attenuated influenza virus vector expressing the SARS-CoV-2 receptor-binding domain (RBD), became the first mucosal COVID-19 vaccine to receive emergency use authorization in December 2022 [71]. India’s iNCOVACC (BBV154), an adenovirus-vectored intranasal vaccine, also received emergency use authorization for booster doses [72]. While these represent milestones for the field, their modest efficacy—the dNS1-RBD vaccine showed approximately 28% efficacy against symptomatic infection—and uncertain durability highlight the challenges of mucosal vaccine development [73].

Adenovirus-vectored intranasal vaccines have demonstrated particularly robust immunogenicity in both preclinical and clinical settings. The ChAd-SARS-CoV-2-S vaccine, based on a chimpanzee adenoviral vector, was shown to prevent sequential transmission of SARS-CoV-2 to unvaccinated hamsters when administered intranasally, providing direct evidence that mucosal vaccination can reduce viral shedding and transmission [39]. Similarly, mucosal adenoviral vaccine boosting in non-human primates elicited durable mucosal IgA responses and prevented infection with the XBB.1.16 variant [39,40]. These data provide a rationale for using intranasal adenoviral vaccines as booster doses following primary systemic vaccination.

Nanoparticle-based intranasal delivery systems are also under development. Silica-based nanoparticles functionalized with mucoadhesive polymers have been designed to enhance antigen retention at the nasal mucosa, prolonging exposure to immune cells and improving the induction of mucosal IgA [74]. These platforms induce sustained systemic and neutralizing antibody responses in preclinical models [74].

### 4.2. Oral Vaccination

Oral vaccination targets the GALT, including Peyer’s patches and isolated lymphoid follicles, and is particularly effective at inducing SIgA responses in the gastrointestinal tract [1,16]. The oral route is the most well-established mucosal vaccination approach, with several licensed vaccines including oral polio vaccine (OPV), oral rotavirus vaccines, oral cholera vaccines, and the oral typhoid vaccine [11,75]. The primary advantages of oral vaccination include ease of administration, suitability for mass immunization campaigns, and the induction of gut-associated mucosal immunity [11].

However, oral vaccine development faces significant challenges, including antigen degradation by gastric acid and digestive enzymes, dilution effects in the large volume of the gastrointestinal tract, and the strong tolerogenic environment of the gut mucosa [13,76]. Various strategies have been developed to overcome these barriers, including encapsulation of antigens in acid-resistant particles, use of enteric-coated formulations, and exploitation of live attenuated vectors that can survive and replicate in the gut [77]. The route of oral delivery of immunomodulatory proteins, such as bovine lactoferrin, influences both mucosal and systemic immune responses in mice, underscoring the importance of formulation design in oral vaccine strategies [78].

Engineered bacterial platforms represent a new strategy for oral mucosal immunization. Probiotic bacteria, particularly *Escherichia coli* Nissle 1917 (EcN), can be genetically engineered to display vaccine antigens on their surface or secrete therapeutic proteins directly into the gut lumen. EcN engineered to express the SARS-CoV-2 spike RBD on its surface induced systemic IgG and mucosal IgA responses upon oral administration in mice, with antibody levels comparable to those from intramuscular mRNA vaccination [14]. Outer membrane vesicles (OMVs) released by these bacteria also facilitated nanobody translocation to distant organs, suggesting dual therapeutic and immunization potential [14].

The concept of programmable antigen release from bacterial vectors has been further advanced through the development of inducible lytic circuits. A probiotic-based oral vaccine system has been developed featuring dual-antigen ferritin arrays and an arabinose-inducible bacterial lysis mechanism; upon lysis, the released antigen-decorated nanoparticles efficiently traversed the intestinal barrier via M-cell targeting and activated mucosal dendritic cells, eliciting robust ${\mathrm{CD8}}^{+}$ and ${\mathrm{CD4}}^{+}$ T cell responses and providing therapeutic efficacy in melanoma models [15]. Similarly, recombinant Lactococcus lactis expressing *Helicobacter pylori* urease antigens has shown protective efficacy in preclinical models, with oral administration inducing antigen-specific sIgA and Th1/Th17 cellular immune responses that prevented bacterial colonization in 70% of mice [79].

A critical technical challenge for live bacterial mucosal vaccines is the efficient export of recombinant antigens from the bacterial cytoplasm to the cell surface or extracellular medium. The bioengineering of the flagellar type III secretion system (FT3SS) in *E. coli* has provided a solution, achieving a 24-fold improvement in secretion of heterologous proteins through iterative strain engineering and modular plasmid design [80]. Improving secretion efficiency will be critical for the clinical translation of live bacterial mucosal vaccines.

### 4.3. Sublingual Vaccination

Sublingual vaccination, in which antigens are applied to the floor of the mouth beneath the tongue, offers an alternative to intranasal and oral routes [81,82]. The sublingual mucosa is highly permeable, well-vascularized, and contains a rich population of antigen-presenting cells, making it an efficient site for antigen uptake and immune induction [81]. Sublingual vaccination avoids the safety concerns of intranasal delivery, such as the potential for vaccine components to access the central nervous system via the olfactory nerve, and does not expose antigens to gastrointestinal degradation [82,83].

Sublingual immunization has been shown to induce both mucosal and systemic immune responses, including SIgA in the respiratory and intestinal tracts [81,82]. However, the induction of optimal mucosal immunity by this route requires appropriate adjuvants, as sublingual delivery of antigen alone is often insufficient to overcome mucosal tolerance [62]. As noted in Section 3.3, sublingual delivery of the STING agonist 3′3′-cGAMP promoted both systemic and mucosal immunity against anthrax toxins [62].

An important study revealed that neutrophils negatively regulate the induction of mucosal IgA responses after sublingual immunization [84]. Neutrophils recruited to the sublingual mucosa after vaccination actively suppress IgA class switching [84]. A supplementation approach was subsequently developed to overcome neutrophil-mediated suppression and enhance mucosal IgA induction [85]. Targeting neutrophil activity may thus broadly improve mucosal vaccine efficacy across delivery routes.

### 4.4. Viral Vector-Based Mucosal Delivery

Viral vectors combine targeted antigen expression with the intrinsic immunogenicity of viral particles, making them well suited for mucosal delivery [86,87]. Platforms developed for intranasal delivery include adenoviruses, vesicular stomatitis virus (VSV), measles virus (MeV), mumps virus (MuV), Newcastle disease virus, and parainfluenza virus [87,88].

A methyltransferase-defective VSV-based SARS-CoV-2 vaccine provided complete protection against infection in hamsters [89]. Building on this work, intranasal SARS-CoV-2 vaccine candidates were developed based on the attenuated MeV and MuV Jeryl Lynn vaccine strains, expressing the stabilized prefusion spike proteins of multiple SARS-CoV-2 variants [90]. Intranasal immunization with these monovalent or trivalent vaccines induced high levels of neutralizing antibodies that efficiently neutralized Omicron subvariants including XBB.1.5, EG.5, and JN.1, providing complete protection against these variants in hamster challenge models [90].

Most recently, safe SARS-CoV-2 Omicron JN.1-based live attenuated vaccine candidates were developed as intranasal bivalent vaccines targeting both SARS-CoV-2 and respiratory syncytial virus (RSV), demonstrating the versatility of this platform for multivalent respiratory mucosal vaccination [91]. These results illustrate the feasibility of multivalent mucosal vaccines targeting multiple respiratory pathogens with a single intranasal administration.

Vector selection requires balancing pre-existing immunity in the target population, transgene capacity, replication competence in mucosal tissues, and vector immunogenicity [86,87]. Replication-competent vectors, such as live attenuated measles and mumps viruses, offer the advantage of antigen amplification at mucosal sites, potentially enhancing the magnitude and durability of immune responses [90]. However, safety considerations, particularly for immunocompromised individuals, may favor the use of replication-deficient vectors such as adenoviruses. Developing vectors with improved safety, reduced susceptibility to pre-existing immunity, and enhanced mucosal tropism remains a priority.

Reverse genetics systems now enable the engineering of attenuated viruses with defined safety mutations. For example, mutations in viral methyltransferases and deletions of virulence genes can be precisely introduced to generate vaccine candidates that are sufficiently attenuated for safe mucosal administration while retaining immunogenicity [89,91]. This rational design approach accelerates development compared with empirical attenuation methods.

## 5. Regulation of Mucosal Immune Responses: Implications for Vaccine Design

The efficacy of mucosal vaccines depends not only on adjuvant and delivery choices but also on the host immune regulatory networks that shape vaccine responses.

### 5.1. Role of Innate Immune Cells

Innate immune cells initiate and regulate mucosal vaccine responses. Dendritic cells bridge innate and adaptive immunity at mucosal surfaces by capturing antigens, producing polarizing cytokines, and migrating to draining lymph nodes to activate naive T cells [92,93]. Different DC subsets contribute distinct functions to mucosal vaccine responses: conventional type 1 DCs (cDC1, including CD8alpha+ DCs) promote Th1 and cytotoxic T lymphocyte responses, while conventional type 2 DCs (cDC2, including CD11b+ DCs) are important for Th2 and Th17 polarization [94].

Neutrophils also regulate mucosal vaccine responses. As discussed in Section 4.3, neutrophils negatively regulate mucosal IgA responses after sublingual immunization [84]. Neutrophils recruited to the vaccination site can suppress IgA class switching through mechanisms that may involve the release of serine proteases, including elastase, and the production of immunosuppressive mediators [69,84]. CT promotes a balanced Th1/Th2/Th17 response by acting through Gsα in CD11b+ DCs independently of IL-12 and IL-17, identifying this DC subset as a key target for mucosal adjuvant action [95].

Macrophages in the lamina propria, for example, contribute to antigen sampling and the production of cytokines such as IL-10 and TGF-beta that promote IgA class switching and maintain mucosal homeostasis [92,93]. Innate lymphoid cells (ILCs), particularly ILC3, have been recognized as important producers of IL-22 and IL-17 at mucosal surfaces, contributing to epithelial barrier defense and the regulation of commensal bacteria [96]. Targeting these innate cell populations through adjuvant design may improve the precision of mucosal vaccine responses.

Mast cells and eosinophils also participate in mucosal immune responses, although their roles in vaccine-induced immunity are less well characterized. These cell types can serve as antigen-presenting cells under certain conditions and can produce cytokines and mediators that modulate adaptive immune responses [97]. How different adjuvants engage these cellular networks remains an open question with direct implications for mucosal vaccine design.

### 5.2. T Helper Cell Subsets and Mucosal Immunity

The balance of ${\mathrm{CD4}}^{+}$ T helper cell subsets shapes the quality of mucosal vaccine responses [97,98]. Th1 cells produce IFN-γ and promote opsonizing IgG2a responses against intracellular pathogens. Th2 cells produce IL-4, IL-5, and IL-13, supporting humoral immunity and SIgA induction [26,97]. Th17 cells produce IL-17 and IL-22, contributing to mucosal barrier defense and antimicrobial peptide induction [96].

Both Th1 and Th2 cells are required for eosinophil- and neutrophil-associated airway inflammatory responses, indicating that subset interplay determines mucosal effector mechanisms [99]. The route of cytokine delivery can selectively modulate T helper cell polarization: oral but not parenteral IL-12 redirects Th2-type responses to an oral vaccine without altering mucosal IgA [57]. Combining appropriate adjuvants with specific delivery routes can thus achieve the desired Th cell balance for mucosal and systemic protection.

As discussed in Section 2.2, Tfh cells are key regulators of IgA class switching in mucosal tissues [30]. Tfh cells provide essential help to B cells in germinal centers through the production of IL-21 and through CD40L-mediated signals. Mucosal vaccination generates Tfh cells in mucosal lymphoid tissues, and the magnitude of Tfh responses correlates with high-affinity, long-lived SIgA production [30,31].

Regulatory T cells (Tregs) add further complexity to mucosal vaccine responses. Tregs prevent excessive inflammation and maintain homeostasis at mucosal surfaces [100]. While Tregs are essential for preventing autoimmunity and inflammatory bowel disease, their activity at mucosal sites can also dampen vaccine-induced immune responses. Potent mucosal adjuvants such as CT and CpG ODN can overcome Treg-mediated suppression to enable productive mucosal immune responses [45,58].

### 5.3. Microbiota and Mucosal Vaccine Efficacy

The commensal microbiota shapes mucosal immune development and modulates vaccine responses [100,101]. The gut microbiota promotes the development of the mucosal immune system, including the induction of IgA-producing plasma cells, the expansion of regulatory T cells, and the education of innate immune cells [101,102]. Perturbations of the microbiota, such as those caused by antibiotic use, malnutrition, or environmental factors, can significantly alter mucosal immune responses and vaccine efficacy [103,104].

We demonstrated that broad-spectrum and Gram-negative-targeting antibiotics differentially regulate antibody isotype responses to injected vaccines, shifting the balance between IgG and IgA depending on the antibiotic class [105]. Given the widespread use of concurrent antibiotics and vaccination, these results suggest that antibiotic timing and spectrum should be considered in vaccine administration guidelines.

The microbiota also influences mucosal vaccine responses through its effects on innate immune signaling and epithelial barrier function. Commensal bacteria produce metabolites, including short-chain fatty acids and bile acid derivatives, that modulate immune cell function and can enhance or suppress vaccine responses depending on the context [106,107]. Optimizing vaccine efficacy across populations with diverse microbiome compositions will require a better understanding of these microbiota-immune interactions.

Beyond shaping vaccine responses, the gut microbiota can be actively modulated using engineered bacteria. Orally administered engineered EcN expressing anti-inflammatory molecules from Faecalibacterium prausnitzii improved survival and cardiac function after myocardial infarction in preclinical models, demonstrating that engineered probiotics can reshape host–microbiome immune interactions beyond the gut [108].

## 6. Current Landscape of Approved and Clinical-Stage Mucosal Vaccines

### 6.1. Licensed Mucosal Vaccines

Despite decades of research, only a limited number of mucosal vaccines have been licensed for human use. These include the oral polio vaccine (OPV), oral rotavirus vaccines (RotaTeq, Rotarix, Rotavac), oral cholera vaccines (Dukoral, Shanchol, Euvichol), the oral typhoid vaccine (Vivotif), and the intranasal live attenuated influenza vaccine (LAIV, marketed as FluMist/Fluenz) [11,75,109]. All rely on live attenuated pathogens or inactivated whole organisms that retain sufficient immunogenicity to induce mucosal responses without exogenous adjuvants.

LAIV induces both local mucosal IgA and systemic immune responses. Its efficacy in children is well documented, although performance in adults and the elderly is more variable [109]. A notable advantage of LAIV is the induction of broadly cross-reactive mucosal IgA that provides partial protection against antigenically drifted influenza strains [110]. OPV induces sterilizing gut immunity that prevents viral shedding and transmission, a property that has been central to the global polio eradication effort [11]. The success of OPV demonstrates that mucosal vaccines can not only prevent disease but also interrupt pathogen transmission—a goal now increasingly pursued for respiratory pathogens such as SARS-CoV-2 and influenza.

The small number of licensed mucosal vaccines reflects several persistent barriers to clinical translation. First, validated mucosal correlates of protection (CoP) remain undefined for most pathogens. For injectable vaccines, serum neutralizing antibody titers serve as the accepted CoP. No equivalent consensus exists for mucosal vaccines, where the relative contributions of SIgA titer, $T_{\mathrm{RM}}$ frequency, and functional antibody activity to protection remain unclear. [73,111]. This gap complicates regulatory approval, as sponsors cannot demonstrate efficacy using established immune endpoints. Second, no mucosal adjuvant for subunit vaccines has yet received regulatory approval for human use. The most potent mucosal adjuvants, CT and LT, are too toxic for clinical application, and while safer derivatives such as dmLT are advancing through clinical trials, none has been licensed [54,55]. Without approved mucosal adjuvants, subunit-based mucosal vaccines cannot be developed. Third, formulation instability poses a practical barrier: antigens delivered to mucosal surfaces face enzymatic degradation, dilution, and rapid mucociliary clearance, requiring specialized formulations that add cost and complexity [12,112]. Fourth, clinical trial design for mucosal vaccines is hampered by the lack of standardized methods for collecting and analyzing mucosal specimens. Nasal washes, bronchoalveolar lavage, and saliva samples are inherently variable, making it difficult to compare results across studies or establish regulatory benchmarks [73,113]. The modest efficacy of the first approved intranasal COVID-19 vaccine, dNS1-RBD (approximately 28% against symptomatic infection) [71], illustrates how these challenges converge in practice: without optimized adjuvants, validated mucosal endpoints, and standardized immune monitoring, even promising mucosal vaccine platforms may yield disappointing clinical results.

### 6.2. COVID-19 Mucosal Vaccine Pipeline

The COVID-19 pandemic catalyzed an unprecedented expansion of the mucosal vaccine pipeline [73,114]. The Chinese intranasal dNS1-RBD vaccine, based on a live attenuated influenza virus vector with a deleted NS1 gene, became the first mucosal COVID-19 vaccine to receive emergency use authorization in China in December 2022 [71]. A phase III trial involving over 31,000 volunteers demonstrated approximately 28% efficacy against symptomatic infection, with a safety profile comparable to placebo [71]. The Russian Salnavac, an intranasal formulation of the Sputnik V adenoviral vector, was registered in Russia in April 2022 [115]. In India, the adenovirus-vectored iNCOVACC (BBV154) received emergency use authorization for intranasal booster doses [72]. These approvals, while representing firsts for the field, also exposed the difficulty of achieving robust efficacy through mucosal routes, as efficacy data for some of these vaccines were modest compared with injectable alternatives [73].

A particularly promising strategy to emerge from COVID-19 research is the “prime-pull” or heterologous prime-boost approach, in which systemic priming with an injectable vaccine is followed by mucosal boosting to establish local immunity [116,117]. Intranasal boosting after intramuscular mRNA vaccination converts pre-existing systemic immunity into mucosal IgA responses and $T_{\mathrm{RM}}$ and $B_{\mathrm{RM}}$ cells in the respiratory tract [116,117]. This approach leverages the strengths of both systemic and mucosal vaccination and may be a practical strategy for enhancing mucosal protection in populations already primed by injectable COVID-19 vaccines.

Mechanistic studies have revealed that intranasal boosting with unadjuvanted recombinant spike protein, following intramuscular mRNA priming, elicits protective mucosal immunity by retooling lymph node resident immune cells [117]. Peripheral lymph node-primed B cells migrate to the lung through CXCR3–CXCL9/CXCL10 signaling and differentiate into IgA-secreting plasma cells. Memory ${\mathrm{CD4}}^{+}$ T cells in the lung act as natural adjuvants by inducing chemokines that recruit memory B cells [117]. These findings suggest that mucosal boosting may not require the complex adjuvant formulations typically needed for primary mucosal immunization, as pre-existing systemic immunity provides a foundation on which mucosal responses can be built.

The concept of immune conversion through mucosal boosting is now being extended to other respiratory pathogens. Heterologous prime-boost strategies combining injectable primary vaccination with intranasal boosters are being explored for influenza, RSV, and tuberculosis, aiming to establish respiratory mucosal immunity in populations with pre-existing systemic immunity [110,118,119].

### 6.3. Beyond COVID-19: Emerging Mucosal Vaccine Targets

The momentum from COVID-19 mucosal vaccine development is now being applied to other respiratory and enteric pathogens. RSV, influenza, and tuberculosis are among the highest-priority targets [118,120]. Universal influenza vaccines delivered intranasally, capable of inducing broadly cross-reactive mucosal responses, are a major goal of the field [110]. Mucosal vaccination approaches for tuberculosis, including intranasal Bacillus Calmette-Guérin (BCG) and viral vectored vaccines, are being explored to complement or replace intradermal BCG [119].

In the biodefense arena, mucosal vaccines against anthrax and botulinum toxins have been pursued for decades. Nasal immunization with protective antigen and mucosal adjuvants induces effective mucosal immunity against anthrax, establishing proof of concept for needle-free mucosal biodefense vaccines [63,70].

## 7. Challenges and Future Perspectives

### 7.1. Correlates of Protection for Mucosal Vaccines

As discussed in Section 6.1, the absence of validated mucosal correlates of protection (CoP) is a major barrier to clinical development [73,121]. SIgA has long been considered the primary effector of mucosal immunity, but the relative contributions of mucosal IgA, serum neutralizing antibodies, $T_{\mathrm{RM}}$ cells, and other immune parameters to protection remain unclear and may vary by pathogen [73,122].

Standardized methods for collecting and analyzing mucosal specimens are urgently needed. Nasal washes, bronchoalveolar lavage fluid, saliva, and stool samples are inherently variable, limiting cross-study comparisons [111]. Minimally invasive techniques such as nasal absorption strips and oral fluid collection devices now enable repeated sampling in clinical trials without invasive procedures [111]. Systems serology approaches that measure multiple antibody functions simultaneously, including neutralization, opsonophagocytosis, and complement activation, may provide a more complete picture of mucosal protection than IgA titers alone [121]. Integrating these analytical tools into mucosal vaccine trials will be essential for defining immune parameters that predict protection.

### 7.2. Safety Considerations

Safety is a key concern for mucosal vaccines, particularly those delivered intranasally. The proximity of the nasal cavity to the olfactory nerve and central nervous system raises the risk that vaccine components could access the brain [113,123]. This concern was substantiated when the nasal adjuvant LTK63, a detoxified LT mutant, was associated with transient facial nerve palsy (Bell’s palsy) in a clinical trial, leading to its withdrawal [124]. Subsequent mucosal adjuvant development has prioritized demonstrating the absence of neurotropism.

A second concern is the potential for mucosal vaccines to induce tolerance rather than immunity when antigens are delivered without appropriate adjuvants [13]. The tolerogenic properties of mucosal surfaces, evolved to prevent inflammatory responses to food antigens and commensal microbes, can suppress vaccine immunogenicity. Adjuvant selection and formulation design must therefore ensure that protective responses are reliably induced.

### 7.3. Formulation and Stability

Mucosal vaccine formulations must resist enzymatic degradation, maintain contact with the mucosal epithelium, and preserve immunogenicity throughout manufacturing, storage, and delivery [125,126]. Strategies to meet these requirements include encapsulation in polymeric nanoparticles, incorporation into mucoadhesive gels and films, and formulation as dry powder aerosols [112,127].

Nanoparticle-based systems have shown particular potential. PLGA nanoparticles co-loaded with antigen and Toll-like receptor (TLR) agonists and coated with mucoadhesive chitosan derivatives induced both systemic and mucosal immune responses after intranasal administration [128]. Glycol chitosan-coated PLGA nanoparticles prolonged nasal residence time and enhanced antibody responses compared with uncoated formulations in intranasal hepatitis B vaccination [129]. Oral PLGA nanoparticles co-encapsulating antigen and monophosphoryl lipid A also promoted serum IgG and mucosal IgA [130]. Mucoadhesive formulations designed to penetrate the mucus layer and deliver antigens to the underlying epithelium are also under development [74].

Cold chain independence is critical for mucosal vaccines intended for global deployment. Thermostable formulations such as dry powder aerosols and sublingual tablets could eliminate cold-chain requirements while maintaining potency [125,127].

### 7.4. Future Directions

**mRNA and saRNA platforms.** The success of mRNA-lipid nanoparticle (mRNA-LNP) technology in the COVID-19 pandemic has driven efforts to adapt this platform for mucosal delivery [131,132]. Intranasal mRNA-LNP vaccines induce both systemic and mucosal responses, including SIgA and $T_{\mathrm{RM}}$ cells, in preclinical studies [133], although optimizing LNP stability and uptake in the mucosal environment remains a challenge. Self-amplifying RNA (saRNA) vaccines offer a complementary approach: unlike conventional mRNA, saRNA encodes the viral replicase machinery for intracellular amplification of the mRNA transcript, potentially enabling lower doses and stronger immunogenicity at mucosal surfaces [131]. Combining saRNA with LNP formulations designed for mucosal stability could accelerate the development of mucosal vaccines against emerging pathogens.

**Rational antigen design.** Advances in structural biology and computational immunology enable the design of antigens optimized for mucosal delivery. Structure-based stabilization of viral proteins in their prefusion conformation, as exemplified by the six-proline-stabilized SARS-CoV-2 spike [134], enhances immunogenicity and antibody breadth. Machine learning approaches are also being applied to predict optimal antigen sequences and adjuvant-delivery combinations [135].

**Multivalent mucosal vaccines.** Mucosal platforms can be engineered to target multiple respiratory pathogens simultaneously. SARS-CoV-2-based bivalent vaccines targeting both SARS-CoV-2 and RSV [91] illustrate the feasibility of delivering comprehensive respiratory protection with a single intranasal administration.

**Virus-like particles.** VLPs are self-assembling, non-infectious nanostructures that mimic native virions but lack genomic material [136,137]. Their repetitive surface epitope arrays efficiently cross-link B cell receptors, enabling potent antibody induction at low antigen concentrations, often without exogenous adjuvants [137]. Norovirus VLPs are particularly attractive for oral delivery due to their gastrointestinal stability, mucoadhesive properties, and scalable production in insect cell and plant-based systems [136,138]. Intranasal bivalent norovirus VLP dry powder formulations elicit robust mucosal IgA that correlates with functional blocking activity [139]. The modular nature of VLP platforms enables surface decoration with heterologous antigens via SpyTag/SpyCatcher conjugation or genetic fusion [136], and rational disulfide engineering further enhances thermostability [140]. Phase 2 clinical data from an oral norovirus VLP vaccine confirmed induction of mucosal IgA in nasal, salivary, and fecal samples, with evidence that mucosal IgA serves as a correlate of protection [141].

**Engineered bacterial delivery systems.** Programmable probiotic chassis offer oral administration, in situ antigen production, and direct interfacing with gut-associated lymphoid tissue. Advances in the flagellar type III secretion system [80] and inducible lytic circuits for controlled antigen release [15] are addressing key barriers to clinical translation of live bacterial mucosal vaccines.

**Trained immunity.** Trained immunity, the epigenetic reprogramming of innate immune cells following initial stimulation, may enhance mucosal vaccine responses [92]. Mucosal adjuvants including TLR agonists and β-glucans induce trained immunity in monocytes and macrophages, potentially providing early protection before adaptive responses are established.

**Systems biology integration.** High-dimensional analysis of mucosal immune responses using single-cell transcriptomics, spatial proteomics, and multi-omics approaches will provide insight into the cellular and molecular networks governing mucosal vaccine responses [142,143]. Combined with continued adjuvant and formulation development, these tools position the field for substantial progress in the coming decade.

## 8. Conclusions

This review has surveyed the current state of mucosal vaccine development across adjuvant design, delivery platforms, immune regulation, and clinical translation. First, the mucosal adjuvant landscape has expanded beyond classical enterotoxins. Safer derivatives such as dmLT are now in clinical trials, and new adjuvant classes including STING agonists, combination adjuvant systems, and the elastase inhibition strategy offer distinct mechanisms to induce SIgA from diverse delivery routes [51,54,62,69]. Second, the delivery platform toolkit has grown rapidly. Viral vectors, nanoparticles, mRNA-LNP, VLPs, and engineered bacterial systems each offer specific advantages, and the choice of platform must be matched to the target pathogen, delivery route, and population. Third, the prime-pull strategy, in which systemic priming is followed by mucosal boosting, has emerged as a practical approach to convert existing systemic immunity into mucosal protection [116,117].

Despite this progress, critical barriers persist. No mucosal adjuvant for subunit vaccines has been licensed for human use. Validated mucosal correlates of protection remain undefined, limiting regulatory pathways. Standardized methods for measuring mucosal immune responses in clinical trials are lacking. The modest efficacy of the first approved intranasal COVID-19 vaccines underscores the gap between preclinical promise and clinical reality [71,73].

Looking ahead, several priorities will shape the field: establishing mucosal immune endpoints accepted by regulatory agencies, developing thermostable, needle-free formulations for global deployment, and integrating systems-biology approaches to define immune parameters that predict mucosal protection. The convergence of advances in adjuvant biology, delivery engineering, and clinical immunology provides a strong foundation for the next generation of mucosal vaccines that can prevent infection at the surfaces where most pathogens first enter the host.

## Data Availability

No new data were created or analyzed in this study. Data sharing is not applicable.

## Abbreviations

The following abbreviations are used in this manuscript:

BALT	Bronchus-associated lymphoid tissue
CMIS	Common mucosal immune system
CoP	Correlates of protection
CpG ODN	CpG oligodeoxynucleotides
CT	Cholera toxin
DCs	Dendritic cells
GALT	Gut-associated lymphoid tissue
GM-CSF	Granulocyte-macrophage colony-stimulating factor
ILCs	Innate lymphoid cells
LAIV	Live attenuated influenza vaccine
LNP	Lipid nanoparticle
LT	Heat-labile enterotoxin
mRNA-LNP	mRNA-lipid nanoparticle
NALT	Nasopharynx-associated lymphoid tissue
pIgR	Polymeric immunoglobulin receptor
PLGA	Poly(lactic-co-glycolic acid)
RSV	Respiratory syncytial virus
saRNA	Self-amplifying RNA
SC	Secretory component
SIgA	Secretory immunoglobulin A
STING	Stimulator of interferon genes
VLPs	Virus-like particles
VSV	Vesicular stomatitis virus

## References

[1] Holmgren J., Czerkinsky C. (2005). Mucosal immunity and vaccines. Nat. Med..

[2] Boyaka P., McGhee J., Mestecky J., Strober W., Russell M.W., Cheroutre H., Lambrecht B.N., Kelsall B.L. (2015). Mucosal Vaccines: An Overview. Mucosal Immunology.

[3] Corthesy B. (2013). Multi-faceted functions of secretory IgA at mucosal surfaces. Front. Immunol..

[4] Boullier S., Tanguy M., Kadaoui K.A., Caubet C., Sansonetti P., Corthésy B., Phalipon A. (2009). Secretory IgA-mediated neutralization of *Shigella flexneri* prevents intestinal tissue destruction by down-regulating inflammatory circuits. J. Immunol..

[5] Neutra M., Kozlowski P. (2006). Mucosal vaccines: The promise and the challenge. Nat. Rev. Immunol..

[6] Boyaka P. (2017). Inducing Mucosal IgA: A Challenge for Vaccine Adjuvants and Delivery Systems. J. Immunol..

[7] Azzi L., Dalla Gasperina D., Veronesi G., Shallak M., Ietto G., Iovino D., Baj A., Gianfagna F., Maurino V., Focosi D. (2022). Mucosal immune response in BNT162b2 COVID-19 vaccine recipients. eBioMedicine.

[8] Lasrado N., Rowe M., McMahan K., Hachmann N.P., Miller J., Jacob-Dolan C., Liu J., Verrette B., Gotthardt K.A., Ty D.M. (2024). SARS-CoV-2 XBB.1.5 mRNA booster vaccination elicits limited mucosal immunity. Sci. Transl. Med..

[9] Wang Q., Iketani S., Li Z., Liu L., Guo Y., Huang Y., Bowen A.D., Liu M., Wang M., Yu J. (2023). Alarming antibody evasion properties of rising SARS-CoV-2 BQ and XBB subvariants. Cell.

[10] Morens D., Taubenberger J., Fauci A. (2023). Rethinking next-generation vaccines for coronaviruses, influenzaviruses, and other respiratory viruses. Cell Host Microbe.

[11] Levine M., Dougan G. (1998). Optimism over vaccines administered via mucosal surfaces. Lancet.

[12] Lycke N. (2012). Recent progress in mucosal vaccine development: Potential and limitations. Nat. Rev. Immunol..

[13] Lamichhane A., Azegami T., Kiyono H. (2014). The mucosal immune system for vaccine development. Vaccine.

[14] Kamble N., Thomas S., Madaan T., Ehsani N., Sange S., Tucker K., Muhumure A., Kunkler S., Kotagiri N. (2025). Engineered bacteria as an orally administered anti-viral treatment and immunization system. Gut Microbes.

[15] Yue Y., Xin Q., Zhu Y., Zhu D., Zhang B., Yan X., Jiang B. (2025). Probiotic-based oral vaccine mucosal delivery system enabling genetically encoded dual-antigen arrays. Nat. Commun..

[16] Mowat A. (2003). Anatomical basis of tolerance and immunity to intestinal antigens. Nat. Rev. Immunol..

[17] Kiyono H., Fukuyama S. (2004). NALT- versus PEYER’S-patch-mediated mucosal immunity. Nat. Rev. Immunol..

[18] Pabst R. (2003). Bronchus-associated lymphoid tissue. An entry site for antigens for successful mucosal vaccinations?. Am. J. Respir. Cell Mol. Biol..

[19] Hase K., Kawano K., Nochi T., Pontes G.S., Fukuda S., Ebisawa M., Kadokura K., Tobe T., Fujimura Y., Kawano S. (2009). Uptake through glycoprotein 2 of FimH^+^ bacteria by M cells initiates mucosal immune response. Nature.

[20] Boyaka P.N., Wright P.F., Marinaro M., Kiyono H., Johnson J.E., Gonzales R.A., Ikizler M.R., Werkhaven J.A., Jackson R.J., Fujihashi K. (2000). Human nasopharyngeal-associated lymphoreticular tissues: Functional analysis of subepithelial and intraepithelial B and T cells from adenoids and tonsils. Am. J. Pathol..

[21] Czerkinsky C., Holmgren J. (2012). Mucosal delivery routes for optimal immunization: Targeting immunity to the right tissues. Mucosal Vaccines.

[22] Zuercher A.W., Coffin S.E., Thurnheer M.C., Fundova P., Cebra J.J. (2002). Nasal-associated lymphoid tissue is a mucosal inductive site for virus-specific humoral and cellular immune responses. J. Immunol..

[23] McDermott M., Bienenstock J. (1979). Evidence for a common mucosal immunologic system. I. Migration of B immunoblasts into intestinal, respiratory, and genital tissues. J. Immunol..

[24] Kaetzel C. (2005). The polymeric immunoglobulin receptor: Bridging innate and adaptive immune responses at mucosal surfaces. Immunol. Rev..

[25] Macpherson A., Koller Y., McCoy K. (2015). The bilateral responsiveness between intestinal microbes and IgA. Trends Immunol..

[26] Beagley K.W., Eldridge J.H., Lee F., Kiyono H., Everson M.P., Koopman W.J., Hirano T., Kishimoto T., McGhee J.R. (1989). Interleukins and IgA synthesis. Human and murine interleukin 6 induce high rate IgA secretion in IgA-committed B cells. J. Exp. Med..

[27] Cerutti A. (2008). The regulation of IgA class switching. Nat. Rev. Immunol..

[28] Boyaka P.N., Marinaro M., Jackson R.J., Menon S., Kiyono H., Jirillo E., McGhee J.R. (1999). IL-12 is an effective adjuvant for induction of mucosal immunity. J. Immunol..

[29] Boyaka P.N., Marinaro M., Vancott J.L., Takahashi I., Fujihashi K., Yamamoto M., van Ginkel F.W., Jackson R.J., Kiyono H., McGhee J.R. (1999). Strategies for mucosal vaccine development. Am. J. Trop. Med. Hyg..

[30] Crotty S. (2019). T follicular helper cell biology: A decade of discovery and diseases. Immunity.

[31] Cao A.T., Yao S., Gong B., Nurieva R.I., Elson C.O., Cong Y. (2015). Interleukin (IL)-21 promotes intestinal IgA response to microbiota. Mucosal Immunol..

[32] He B., Xu W., Santini P.A., Polydorides A.D., Chiu A., Estrella J., Shan M., Chadburn A., Villanacci V., Plebani A. (2007). Intestinal bacteria trigger T cell-independent immunoglobulin A_2_ class switching by inducing epithelial-cell secretion of the cytokine APRIL. Immunity.

[33] Tsuji M., Suzuki K., Kitamura H., Maruya M., Kinoshita K., Ivanov I.I., Itoh K., Littman D.R., Fagarasan S. (2008). Requirement for lymphoid tissue-inducer cells in isolated follicle formation and T cell-independent immunoglobulin A generation in the gut. Immunity.

[34] Schenkel J., Masopust D. (2014). Tissue-resident memory T cells. Immunity.

[35] Szabo P., Miron M., Farber D. (2019). Location, location, location: Tissue resident memory T cells in mice and humans. Sci. Immunol..

[36] Mueller S., Mackay L. (2016). Tissue-resident memory T cells: Local specialists in immune defence. Nat. Rev. Immunol..

[37] Zheng M., Wakim L. (2022). Tissue resident memory T cells in the respiratory tract. Mucosal Immunol..

[38] Ying B., Darling T.L., Desai P., Liang C.-Y., Dmitriev I.P., Soudani N., Bricker T., Kashentseva E.A., Harastani H., Raju S. (2024). Mucosal vaccine-induced cross-reactive CD8^+^ T cells protect against SARS-CoV-2 XBB.1.5 respiratory tract infection. Nat. Immunol..

[39] Gagne M., Flynn B.J., Andrew S.F., Marquez J., Flebbe D.R., Mychalowych A., Lamb E., Davis-Gardner M.E., Burnett M.R., Serebryannyy L.A. (2024). Mucosal adenovirus vaccine boosting elicits IgA and durably prevents XBB.1.16 infection in nonhuman primates. Nat. Immunol..

[40] McMahan K., Wegmann F., Aid M., Sciacca M., Liu J., Hachmann N.P., Miller J., Jacob-Dolan C., Powers O., Hope D. (2024). Mucosal boosting enhances vaccine protection against SARS-CoV-2 in macaques. Nature.

[41] Allie S.R., Bradley J.E., Mudunuru U., Schultz M.D., Graf B.A., Lund F.E., Randall T.D. (2019). The establishment of resident memory B cells in the lung requires local antigen encounter. Nat. Immunol..

[42] MacLean A., Richmond N., Koneva L., Attar M., Medina C., Thornton E., Gomes A., El-Turabi A., Bachmann M., Rijal P. (2022). Secondary influenza challenge triggers resident memory B cell migration and rapid relocation to boost antibody secretion at infected sites. Immunity.

[43] Iwasaki A., Omer S. (2020). Why and how vaccines work. Cell.

[44] Lapuente D., Fuchs J., Willar J., Antão A.V., Eberlein V., Uhlig N., Issmail L., Schmidt A., Oltmanns F., Peter A.S. (2021). Protective mucosal immunity against SARS-CoV-2 after heterologous systemic prime-mucosal boost immunization. Nat. Commun..

[45] Freytag L., Clements J. (2005). Mucosal adjuvants. Vaccine.

[46] Spangler B. (1992). Structure and function of cholera toxin and the related *Escherichia coli* heat-labile enterotoxin. Microbiol. Rev..

[47] Lycke N. (1997). The mechanism of cholera toxin adjuvanticity. Res. Immunol..

[48] Xu-Amano J., Kiyono H., Jackson R.J., Staats H.F., Fujihashi K., Burrows P.D., Elson C.O., Pillai S., McGhee J.R. (1993). Helper T cell subsets for immunoglobulin A responses: Oral immunization with tetanus toxoid and cholera toxin as adjuvant selectively induces Th2 cells in mucosa associated tissues. J. Exp. Med..

[49] Marinaro M., Staats H.F., Hiroi T., Jackson R.J., Coste M., Boyaka P.N., Okahashi N., Yamamoto M., Kiyono H., Bluethmann H. (1995). Mucosal adjuvant effect of cholera toxin in mice results from induction of T helper 2 (Th2) cells and IL-4. J. Immunol..

[50] Takahashi I., Marinaro M., Kiyono H., Jackson R.J., Nakagawa I., Fujihashi K., Hamada S., Clements J.D., Bost K.L., McGhee J.R. (1996). Mechanisms for mucosal immunogenicity and adjuvancy of *Escherichia coli* labile enterotoxin. J. Infect. Dis..

[51] Boyaka P.N., Ohmura M., Fujihashi K., Koga T., Yamamoto M., Kweon M.-N., Takeda Y., Jackson R.J., Kiyono H., Yuki Y. (2003). Chimeras of labile toxin one and cholera toxin retain mucosal adjuvanticity and direct Th cell subsets via their B subunit. J. Immunol..

[52] Hagiwara Y., Kawamura Y.I., Kataoka K., Rahima B., Jackson R.J., Komase K., Dohi T., Boyaka P.N., Takeda Y., Kiyono H. (2006). A second generation of double mutant cholera toxin adjuvants: Enhanced immunity without intracellular trafficking. J. Immunol..

[53] Pizza M., Giuliani M., Fontana M., Monaci E., Douce G., Dougan G., Mills K., Rappuoli R., Del Giudice G. (2001). Mucosal vaccines: Non toxic derivatives of LT and CT as mucosal adjuvants. Vaccine.

[54] Norton E.B., Lawson L.B., Freytag L.C., Clements J.D. (2011). Characterization of a mutant *Escherichia coli* heat-labile toxin, LT(R192G/L211A), as a safe and effective oral adjuvant. Clin. Vaccine Immunol..

[55] Clements J., Norton E. (2018). The mucosal vaccine adjuvant LT(R192G/L211A) or dmLT. mSphere.

[56] Boyaka P., McGhee J. (2001). Cytokines as adjuvants for the induction of mucosal immunity. Adv. Drug Deliv. Rev..

[57] Marinaro M., Boyaka P.N., Jackson R.J., Finkelman F.D., Kiyono H., Jirillo E., McGhee J.R. (1999). Use of intranasal IL-12 to target predominantly Th1 responses to nasal and Th2 responses to oral vaccines given with cholera toxin. J. Immunol..

[58] Fukuiwa T., Sekine S., Kobayashi R., Suzuki H., Kataoka K., Gilbert R.S., Kurono Y., Boyaka P.N., Krieg A.M., McGhee J.R. (2008). A combination of Flt3 ligand cDNA and CpG ODN as nasal adjuvant elicits NALT dendritic cells for prolonged mucosal immunity. Vaccine.

[59] Fujihashi K., Boyaka P. (2014). Mucosal adjuvants for vaccines to control upper respiratory infections in the elderly. Exp. Gerontol..

[60] Burdette D.L., Monroe K.M., Sotelo-Troha K., Iwig J.S., Eckert B., Hyodo M., Hayakawa Y., Vance R.E. (2011). STING is a direct innate immune sensor of cyclic di-GMP. Nature.

[61] Ebensen T., Libanova R., Schulze K., Yevsa T., Morr M., Guzmán C.A. (2011). Bis-(3′,5′)-cyclic dimeric adenosine monophosphate: Strong Th1/Th2/Th17 promoting mucosal adjuvant. Vaccine.

[62] Martin T.L., Jee J., Kim E., Steiner H.E., Cormet-Boyaka E., Boyaka P.N. (2017). Sublingual targeting of STING with 3′3′-cGAMP promotes systemic and mucosal immunity against anthrax toxins. Vaccine.

[63] Duverger A., Jackson R.J., van Ginkel F.W., Fischer R., Tafaro A., Leppla S.H., Fujihashi K., Kiyono H., McGhee J.R., Boyaka P.N. (2006). *Bacillus anthracis* edema toxin acts as an adjuvant for mucosal immune responses to nasally administered vaccine antigens. J. Immunol..

[64] Duverger A., Carré J.-M., Jee J., Leppla S.H., Cormet-Boyaka E., Tang W.-J., Tomé D., Boyaka P.N. (2010). Contributions of edema factor and protective antigen to the induction of protective immunity by *Bacillus anthracis* edema toxin as an intranasal adjuvant. J. Immunol..

[65] Kensil C., Kammer R. (1998). QS-21: A water-soluble triterpene glycoside adjuvant. Expert Opin. Investig. Drugs.

[66] Boyaka P.N., Marinaro M., Jackson R.J., van Ginkel F.W., Cormet-Boyaka E., Kirk K.L., Kensil C.R., McGhee J.R. (2001). Oral QS-21 requires early IL-4 help for induction of mucosal and systemic immunity. J. Immunol..

[67] Marrack P., McKee A., Munks M. (2009). Towards an understanding of the adjuvant action of aluminium. Nat. Rev. Immunol..

[68] HogenEsch H. (2002). Mechanisms of stimulation of the immune response by aluminum adjuvants. Vaccine.

[69] Kim E., Attia Z., Woodfint R.M., Zeng C., Kim S.H., Steiner H.E., Shukla R.K., Liyanage N.P.M., Ghimire S., Li J. (2021). Inhibition of elastase enhances the adjuvanticity of alum and promotes anti-SARS-CoV-2 systemic and mucosal immunity. Proc. Natl. Acad. Sci. USA.

[70] Boyaka P.N., Tafaro A., Fischer R., Leppla S.H., Fujihashi K., McGhee J.R. (2003). Effective mucosal immunity to anthrax: Neutralizing antibodies and Th cell responses following nasal immunization with protective antigen. J. Immunol..

[71] Zhu F., Huang S., Liu X., Chen Q., Zhuang C., Zhao H., Han J., Jaen A.M., Do T.H., Peter J.G. (2023). Safety and efficacy of the intranasal spray SARS-CoV-2 vaccine dNS1-RBD: A multicentre, randomised, double-blind, placebo-controlled, phase 3 trial. Lancet Respir. Med..

[72] Sunagar R., Prasad S.D., Ella R., Vadrevu K.M. (2022). Preclinical evaluation of safety and immunogenicity of a primary series intranasal COVID-19 vaccine candidate (BBV154). Front. Immunol..

[73] Knisely J.M., Buyon L.E., Mandt R., Farkas R., Balasingam S., Bok K., Buchholz U.J., D’souza M.P., Gordon J.L., King D.F.L. (2023). Mucosal vaccines for SARS-CoV-2: Scientific gaps and opportunities-workshop report. npj Vaccines.

[74] Netsomboon K., Bernkop-Schnurch A. (2016). Mucoadhesive vs. mucopenetrating particulate drug delivery. Eur. J. Pharm. Biopharm..

[75] Vela Ramirez J., Sharpe L., Peppas N. (2017). Current state and challenges in developing oral vaccines. Adv. Drug Deliv. Rev..

[76] Walker R. (2005). Considerations for development of whole cell bacterial vaccines to prevent diarrheal diseases in children in developing countries. Vaccine.

[77] Yuki Y., Kiyono H. (2009). Mucosal vaccines: Novel advances in technology and delivery. Expert Rev. Vaccines.

[78] Boyaka P.N., Sfeir R.M., Dubarry M., Rautureau M., Tome D. (2004). The mode of oral bovine lactoferrin administration influences mucosal and systemic immune responses in mice. J. Nutr..

[79] Ni X., Liu Y., Sun M., Jiang Y., Wang Y., Ke D., Guo G., Liu K. (2024). Oral live-carrier vaccine of recombinant *Lactococcus lactis* inducing prophylactic protective immunity against *Helicobacter pylori* infection. Probiotics Antimicrob. Proteins.

[80] Green C., Kamble N., Court E., Bryant O., Hicks M., Lennon C., Fraser G., Wright P., Stafford G. (2019). Engineering the flagellar type III secretion system: Improving capacity for secretion of recombinant protein. Microb. Cell Factories.

[81] Czerkinsky C., Çuburu N., Kweon M.-N., Anjuere F., Holmgren J. (2011). Sublingual vaccination. Hum. Vaccines.

[82] Kweon M. (2011). Sublingual mucosa: A new vaccination route for systemic and mucosal immunity. Cytokine.

[83] Song J.-H., Nguyen H.H., Cuburu N., Horimoto T., Ko S.-Y., Park S.-H., Czerkinsky C., Kweon M.-N. (2008). Sublingual vaccination with influenza virus protects mice against lethal viral infection. Proc. Natl. Acad. Sci. USA.

[84] Jee J., Bonnegarde-Bernard A., Duverger A., Iwakura Y., Cormet-Boyaka E., Martin T.L., E Steiner H., Bachman R.C., Boyaka P.N. (2015). Neutrophils negatively regulate induction of mucosal IgA responses after sublingual immunization. Mucosal Immunol..

[85] Rowe J.C., Attia Z., Kim E., Cormet-Boyaka E., Boyaka P.N. (2019). A novel supplementation approach to enhance host response to sublingual vaccination. Sci. Rep..

[86] Afkhami S., Yao Y., Xing Z. (2016). Methods and clinical development of adenovirus-vectored vaccines against mucosal pathogens. Mol. Ther. Methods Clin. Dev..

[87] Dhama K., Dhawan M., Tiwari R., Bin Emran T., Mitra S., Rabaan A.A., Alhumaid S., Al Alawi Z., Al Mutair A. (2022). COVID-19 intranasal vaccines: Current progress, advantages, prospects, and challenges. Hum. Vaccines Immunother..

[88] Sunagar R., Singh A., Kumar S. (2023). SARS-CoV-2: Immunity, challenges with current vaccines, and a novel perspective on mucosal vaccines. Vaccines.

[89] Lu M., Zhang Y., Dravid P., Li A., Zeng C., Kc M., Trivedi S., Sharma H., Chaiwatpongsakorn S., Zani A. (2021). A Methyltransferase-Defective Vesicular Stomatitis Virus-Based SARS-CoV-2 Vaccine Candidate Provides Complete Protection Against SARS-CoV-2 Infection in Hamsters. J. Virol..

[90] Hsu C., Chamblee M., Ye C., Shamseldin M.M., Yoo S.J., Li P., Zhang Y., Liu Y., Hall J.M., Xu J. (2025). Intranasal measles virus- and mumps virus-based SARS-CoV-2 vaccine candidates prevent SARS-CoV-2 infection and transmission. Proc. Natl. Acad. Sci. USA.

[91] Xu J., Chamblee M., Jiang F., Kc M., Hsu C.C., Thongpan I., Chen P., Zhang Y., Chiu C.-T., Shamseldin M.M. (2025). Development of SARS-CoV-2 as a viral vector: A novel intranasal bivalent vaccine for SARS-CoV-2 and RSV. Sci. Adv..

[92] Iwasaki A., Medzhitov R. (2010). Regulation of adaptive immunity by the innate immune system. Science.

[93] Banchereau J., Steinman R. (1998). Dendritic cells and the control of immunity. Nature.

[94] Guilliams M., Ginhoux F., Jakubzick C., Naik S.H., Onai N., Schraml B.U., Segura E., Tussiwand R., Yona S. (2014). Dendritic cells, monocytes and macrophages: A unified nomenclature based on ontogeny. Nat. Rev. Immunol..

[95] Mattsson J., Schön K., Ekman L., Fahlén-Yrlid L., Yrlid U., Lycke N.Y. (2015). Cholera toxin adjuvant promotes a balanced Th1/Th2/Th17 response independently of IL-12 and IL-17 by acting on Gsalpha in CD11b^+^ DCs. Mucosal Immunol..

[96] Aujla S.J., Chan Y.R., Zheng M., Fei M., Askew D.J., Pociask D.A., Reinhart T.A., McAllister F., Edeal J., Gaus K. (2008). IL-22 mediates mucosal host defense against Gram-negative bacterial pneumonia. Nat. Med..

[97] Zhu J., Yamane H., Paul W. (2010). Differentiation of effector CD4 T cell populations. Annu. Rev. Immunol..

[98] Sallusto F. (2016). Heterogeneity of human CD4^+^ T cells against microbes. Annu. Rev. Immunol..

[99] Fischer R., Tomé D., McGhee J.R., Boyaka P.N. (2007). Th1 and Th2 cells are required for both eosinophil- and neutrophil-associated airway inflammatory responses in mice. Biochem. Biophys. Res. Commun..

[100] Belkaid Y., Hand T. (2014). Role of the microbiota in immunity and inflammation. Cell.

[101] Hooper L., Macpherson A. (2010). Immune adaptations that maintain homeostasis with the intestinal microbiota. Nat. Rev. Immunol..

[102] Palm N.W., De Zoete M.R., Cullen T.W., Barry N.A., Stefanowski J., Hao L., Degnan P.H., Hu J., Peter I., Zhang W. (2014). Immunoglobulin A coating identifies colitogenic bacteria in inflammatory bowel disease. Cell.

[103] Hagan T., Cortese M., Rouphael N., Boudreau C., Linde C., Maddur M.S., Das J., Wang H., Guthmiller J., Zheng N.-Y. (2019). Antibiotics-Driven Gut Microbiome Perturbation Alters Immunity to Vaccines in Humans. Cell.

[104] Harris V.C., Haak B.W., Handley S.A., Jiang B., Velasquez D.E., Hykes B.L., Droit L., Berbers G.A., Kemper E.M., van Leeuwen E.M. (2018). Effect of Antibiotic-Mediated Microbiome Modulation on Rotavirus Vaccine Immunogenicity: A Human, Randomized-Control Proof-of-Concept Trial. Cell Host Microbe.

[105] Haile A.F., Woodfint R.M., Kim E., Joldrichsen M.R., Berhe N., Gebreyes W.A., Boyaka P.N. (2021). Broad-Spectrum and Gram-Negative-Targeting Antibiotics Differentially Regulate Antibody Isotype Responses to Injected Vaccines. Vaccines.

[106] Kim M., Qie Y., Park J., Kim C.H. (2016). Gut microbial metabolites fuel host antibody responses. Cell Host Microbe.

[107] Campbell C., McKenney P.T., Konstantinovsky D., Isaeva O.I., Schizas M., Verter J., Mai C., Jin W.-B., Guo C.-J., Violante S. (2020). Bacterial metabolism of bile acids promotes generation of peripheral regulatory T cells. Nature.

[108] Madaan T., Nieman M., Rakheja T., Siddiqui N., Gherardini D., Sertorio M., Kamble N., Thomas S., Hartman A., Koch S. (2025). Modulating cardiac-gut microbiome interactions post-myocardial infarction with engineered bacteria. bioRxiv.

[109] Belshe R.B., Coelingh K., Ambrose C.S., Woo J.C., Wu X. (2010). Efficacy of live attenuated influenza vaccine in children against influenza B viruses by lineage and antigenic similarity. Vaccine.

[110] Nachbagauer R., Feser J., Naficy A., Bernstein D.I., Guptill J., Walter E.B., Berlanda-Scorza F., Stadlbauer D., Wilson P.C., Aydillo T. (2021). A chimeric hemagglutinin-based universal influenza virus vaccine approach induces broad and long-lasting immunity in a randomized, placebo-controlled phase I trial. Nat. Med..

[111] Fischinger S., Fallon J.K., Michell A.R., Broge T., Suscovich T.J., Streeck H., Alter G. (2019). A high-throughput, bead-based, antigen-specific assay to assess the ability of antibodies to induce complement activation. J. Immunol. Methods.

[112] Sudduth E.M., Trautmann-Rodriguez M., Gill N., Bomb K., Fromen C.A. (2023). Aerosol pulmonary immune engineering. Adv. Drug Deliv. Rev..

[113] Lewis D.J.M., Huo Z., Barnett S., Kromann I., Giemza R., Galiza E., Woodrow M., Thierry-Carstensen B., Andersen P., Novicki D. (2009). Transient facial nerve paralysis (Bell’s palsy) following intranasal delivery of a genetically detoxified mutant of *Escherichia coli* heat labile toxin. PLoS ONE.

[114] Dotiwala F., Upadhyay A. (2023). Next generation mucosal vaccine strategy for respiratory pathogens. Vaccines.

[115] Logunov D.Y., Dolzhikova I.V., Shcheblyakov D.V., Tukhvatulin A.I., Zubkova O.V., Dzharullaeva A.S., Kovyrshina A.V., Lubenets N.L., Grousova D.M., Erokhova A.S. (2021). Safety and efficacy of an rAd26 and rAd5 vector-based heterologous prime-boost COVID-19 vaccine: An interim analysis of a randomised controlled phase 3 trial in Russia. Lancet.

[116] Mao T., Israelow B., Peña-Hernández M.A., Suberi A., Zhou L., Luyten S., Reschke M., Dong H., Homer R.J., Saltzman W.M. (2022). Unadjuvanted intranasal spike vaccine elicits protective mucosal immunity against sarbecoviruses. Science.

[117] Kwon D.-I., Mao T., Israelow B., de Sá K.S.G., Dong H., Iwasaki A. (2025). Mucosal unadjuvanted booster vaccines elicit local IgA responses by conversion of pre-existing immunity in mice. Nat. Immunol..

[118] Graham B. (2017). Vaccine development for respiratory syncytial virus. Curr. Opin. Virol..

[119] Jeyanathan M., Afkhami S., Smaill F., Miller M.S., Lichty B.D., Xing Z. (2020). Immunological considerations for COVID-19 vaccine strategies. Nat. Rev. Immunol..

[120] Krammer F., Palese P., Shaw M.L., García-Sastre A., Smith G.J.D., Fouchier R.A.M., Peiris M., Kedzierska K., Doherty P.C., Treanor J. (2018). Influenza. Nat. Rev. Dis. Primers.

[121] Plotkin S. (2010). Correlates of protection induced by vaccination. Clin. Vaccine Immunol..

[122] Havervall S., Marking U., Svensson J., Greilert-Norin N., Bacchus P., Nilsson P., Hober S., Gordon M., Blom K., Klingström J. (2022). Anti-Spike Mucosal IgA Protection against SARS-CoV-2 Omicron Infection. N. Engl. J. Med..

[123] van Riet E., Ainai A., Suzuki T., Hasegawa H. (2012). Mucosal IgA responses in influenza virus infections; thoughts for vaccine design. Vaccine.

[124] Mutsch M., Zhou W., Rhodes P., Bopp M., Chen R.T., Linder T., Spyr C., Steffen R. (2004). Use of the inactivated intranasal influenza vaccine and the risk of Bell’s palsy in Switzerland. N. Engl. J. Med..

[125] Amorij J.P., Hinrichs W.L., Frijlink H.W., Wilschut J.C., Huckriede A. (2010). Needle-free influenza vaccination. Lancet Infect. Dis..

[126] Csaba N., Garcia-Fuentes M., Alonso M. (2009). Nanoparticles for nasal vaccination. Adv. Drug Deliv. Rev..

[127] Ye T., Jiao Z., Li X., He Z., Li Y., Yang F., Zhao X., Wang Y., Huang W., Qin M. (2023). Inhaled SARS-CoV-2 vaccine for single-dose dry powder aerosol immunization. Nature.

[128] Primard C., Poecheim J., Heuking S., Sublet E., Esmaeili F., Borchard G. (2013). Multifunctional PLGA-based nanoparticles encapsulating simultaneously hydrophilic antigen and hydrophobic immunomodulator for mucosal immunization. Mol. Pharm..

[129] Pawar D., Mangal S., Goswami R., Jaganathan K. (2013). Development and characterization of surface modified PLGA nanoparticles for nasal vaccine delivery: Effect of mucoadhesive coating on antigen uptake and immune adjuvant activity. Eur. J. Pharm. Biopharm..

[130] Sarti F., Perera G., Hintzen F., Kotti K., Karageorgiou V., Kammona O., Kiparissides C., Bernkop-Schnürch A. (2011). In vivo evidence of oral vaccination with PLGA nanoparticles containing the immunostimulant monophosphoryl lipid A. Biomaterials.

[131] Pardi N., Hogan M.J., Porter F.W., Weissman D. (2018). mRNA vaccines—A new era in vaccinology. Nat. Rev. Drug Discov..

[132] Chaudhary N., Weissman D., Whitehead K. (2021). mRNA vaccines for infectious diseases: Principles, delivery and clinical translation. Nat. Rev. Drug Discov..

[133] Hartwell B.L., Melo M.B., Xiao P., Lemnios A.A., Li N., Chang J.Y., Yu J., Gebre M.S., Chang A., Maiorino L. (2022). Intranasal vaccination with lipid-conjugated immunogens promotes antigen transmucosal uptake to drive mucosal and systemic immunity. Sci. Transl. Med..

[134] Hsieh C.-L., Goldsmith J.A., Schaub J.M., DiVenere A.M., Kuo H.-C., Javanmardi K., Le K.C., Wrapp D., Lee A.G., Liu Y. (2020). Structure-based design of prefusion-stabilized SARS-CoV-2 spikes. Science.

[135] Ong E., Wang H., Wong M.U., Seetharaman M., Valdez N., He Y. (2020). Vaxign-ML: Supervised machine learning reverse vaccinology model for improved prediction of bacterial protective antigens. Bioinformatics.

[136] Lampinen V., Heinimäki S., Laitinen O.H., Pesu M., Hankaniemi M.M., Blazevic V., Hytönen V.P. (2021). Modular vaccine platform based on the norovirus-like particle. J. Nanobiotechnol..

[137] Mohsen M.O., Zha L., Cabral-Miranda G., Bachmann M.F. (2017). Major findings and recent advances in virus-like particle (VLP)-based vaccines. Semin. Immunol..

[138] Chen Q. (2015). Virus-like particle vaccines for norovirus gastroenteritis. Adv. Virus Res..

[139] Ball J.P., Springer M.J., Ni Y., Finger-Baker I., Martinez J., Hahn J., Suber J.F., DiMarco A.V., Talton J.D., Cobb R.R. (2017). Intranasal delivery of a bivalent norovirus vaccine formulated in an in situ gelling dry powder. PLoS ONE.

[140] Warren C., Galli J.D., Bystol K., O’dOnnell G., Swartz A.R., Dewar E.A., Fulton C.M., Shen P., Gonzalez-Fernandez E., DeWitt L.A. (2025). Stabilization of norovirus GII.3 virus-like particles by rational disulfide engineering. npj Vaccines.

[141] Flitter B.A., Gillard J., Greco S.N., Apkarian M.D., D’aMato N.P., Nguyen L.Q., Neuhaus E.D., Hailey D.C.M., Pasetti M.F., Shriver M. (2025). An oral norovirus vaccine generates mucosal immunity and reduces viral shedding in a phase 2 placebo-controlled challenge study. Sci. Transl. Med..

[142] Melms J.C., Biermann J., Huang H., Wang Y., Nair A., Tagore S., Katsyv I., Rendeiro A.F., Amin A.D., Schapiro D. (2021). A molecular single-cell lung atlas of lethal COVID-19. Nature.

[143] Yao C., Bora S.A., Parimon T., Zaman T., Friedman O.A., Palatinus J.A., Surapaneni N.S., Matusov Y.P., Chiang G.C., Kassar A.G. (2021). Cell-type-specific immune dysregulation in severely ill COVID-19 patients. Cell Rep..

